# From Wearable Sensor Networks to Markerless Motion Capture for Instrumental-Based Biomechanical Risk Assessment in Lifting Activities

**DOI:** 10.3390/s25247427

**Published:** 2025-12-06

**Authors:** Irene Gennarelli, Tiwana Varrecchia, Giorgia Chini, Niki Martinel, Christian Micheloni, Alberto Ranavolo

**Affiliations:** 1 Department of Mathematics, Computer Science and Physics, University of Udine, Via Palladio 8, 33100 Udine, Italy; gennarelli.irene@spes.uniud.it (I.G.); niki.martinel@uniud.it (N.M.); christian.micheloni@uniud.it (C.M.); 2 Department of Occupational and Environmental Medicine, Epidemiology and Hygiene, INAIL—National Institute for Insurance Against Accidents at Work, Via Fontana Candida 1, 00078 Monte Porzio Catone, Italy; t.varrecchia@inail.it (T.V.); g.chini@inail.it (G.C.)

**Keywords:** biomechanical risk assessment, wearable sensor network, markerless motion capture

## Abstract

Manual material handling is one of the leading causes of work-related low-back disorders, and an accurate assessment of the biomechanical risk is essential to support prevention strategies. Despite workers’ interest in wearable sensor networks for quantifying exposure metrics, these systems still present several limitations, including potential interference with natural movements and workplaces, and concerns about durability and cost-effectiveness. For these reasons, alternative motion capture methods are being explored. Among them, completely markerless (ML) technologies are being increasingly applied in ergonomics. This study aimed to compare a wearable sensor network and an ML system in the evaluation of lifting tasks, focusing on the variables and multipliers used to compute the recommended weight limit (RWL) and the lifting index (LI) according to the revised NIOSH lifting equation. We hypothesized that ML systems equipped with multiple cameras may provide reliable and consistent estimations of these kinematic variables, thereby improving risk assessments. We also assumed that these ML approaches could represent valuable input for training AI algorithms capable of automatically classifying the biomechanical risk level. Twenty-eight workers performed standardized lifts under three risk conditions. The results showed significant differences between wearable sensor networks and ML systems for most measures, except at a low risk (LI = 1). Nevertheless, ML consistently showed a closer agreement with reference benchmarks and a lower variability. In terms of the automatic classification performance, ML–based kinematic variables yielded accuracy levels comparable to those obtained with the wearable system. These findings highlight the potential of ML approaches to deliver accurate, repeatable, and cost-effective biomechanical risk assessments, particularly in demanding lifting tasks.

## 1. Introduction

Marker-based methods, such as wearable sensor networks composed of inertial measurement units (IMUs), sensorized insoles and shoes, and surface electromyography (sEMG) sensors, allow for both post-processing and real-time biomechanical risk assessments during the execution of manual material handling (MMH) activities directly in the workplace [1,2,3,4]. These sensor-based approaches, recently also implemented within a pre-standard [5], make possible the “rating of standard methods” [6,7,8] listed in the international ergonomics standards of the ISO 11228 series, allowing some of their limits to be overcome [9,10,11,12]. Furthermore, in the new occupational scenarios in which MMH activities can be carried out with the aid of human–robot collaboration (HRC) technologies and exoskeletons [13,14,15,16,17], real-time instrumental-based tools for biomechanical risk assessments allow for the control of HRC technologies and enable workers to be quickly alerted with the use of vibrotactile, visual, and auditory feedback stimuli [18,19,20].

Despite the workers’ reported interest in utilizing wearable sensor networks to assess key exposure metrics, there are still multiple barriers that limit their widespread use [21]. They can interfere with the subjects and workplace and have to be accepted by the organizational management [22]. Moreover, these technologies require in-depth manufacturing practices and calibration procedures. Finally, they have to be evaluated from the point of view of sensor durability and the cost–benefit ratio [23]. For the reasons set out above, it is necessary to identify alternative motion capture systems capable of overcoming some of the limitations of wearable devices. Among these, completely markerless (ML) technologies are being used, according to a recent systematic review, in a growing number of studies and publications belonging to the domain of ergonomics [22]. ML motion capture approaches can be capable, by using computer vision algorithms, of estimating most of the input variables necessary to estimate the biomechanical risk level. They use multiple cameras or depth sensors to identify and measure human kinematics without the need for physical markers, sensors, or suits worn by the worker, reducing the complexity of the experimental setup [24,25]. Each of these algorithms relies on a first task in which the human figure is described by a kinematic-based skeletal model, where each node represents a joint and each linked joint pair is represented as a vector, as well as a mesh model, which reconstructs the human body surface [26].

In lifting heavy loads, to prevent the onset of work-related low-back disorders (WLBDs), the level of risk can be computed by calculating the lifting index (LI) by dividing the actual load lifted (L) by the recommended weight limit (RWL) [27,28,29]. The RWL is calculated by using the revised NIOSH (National Institute for Occupational Safety and Health, USA) lifting equation (RNLE) which requires, among the input variables, the horizontal (H) and vertical (V) locations, the vertical travel distance (D), and the asymmetry angle (A). These kinematic variables can be estimated with the use of wearable sensor networks or by using three-dimensional human movement algorithms for multi-view ML motion capture [25]. These approaches require the use of specific cameras to be positioned in the workplace to monitor the movement of the worker. In addition, the quantitative data generated by wearable and ML systems can be leveraged to train and validate artificial intelligence models for automated motion analyses and ergonomic risk classification [30]. Additionally, beyond generic artificial intelligence algorithms for movement analyses, recent advances in computer vision now enable the use of skeleton-based action recognition networks, which classify human motion based on 3D skeletal joint sequences rather than raw video frames. For example, the SkelMamba model—a state-space model designed for 68 efficient skeleton-based action recognitions in neurological disorders—has demonstrated its applicability on datasets acquired using multi-cameras [31]. While previous studies have implemented this framework using wearable-based joint data, the present work introduces, for the first time, the integration of the SkelMamba model with ML motion capture data, thereby extending its applicability to contactless motion analysis in occupational ergonomics [32].

Although markerless motion capture systems have shown promise in clinical gait and sports biomechanics, their validity in the occupational context, particularly in evaluating the biomechanical risk, remains largely unexplored. Existing studies have typically evaluated markerless systems in controlled laboratory or sports scenarios using generic kinematic metrics (e.g., joint angles, segment lengths), but few have examined whether these systems can reliably reproduce task-specific ergonomic indicators relevant to occupational risk evaluations, particularly during lifting activities, where small kinematic deviations can lead to substantial differences in the calculated risk levels. It would be particularly useful to understand how data generated by a wearable system and a markerless system can be used to train artificial intelligence algorithms to accurately and precisely estimate risk.

We hypothesized that, during lifting activities, the use of ML systems equipped with a large number of cameras could allow for the reliable and consistent measurement of the kinematic input variables required by the RNLE. We also hypothesized that these kinematic variables extracted from ML systems can be used, as well as those extracted from wearable systems, to train AI algorithms capable of recognizing movement and its associated risk condition. In particular, we aimed to compare the performance of a skeleton-based risk classification network trained on data acquired from both systems, in order to evaluate whether markerless and wearable technologies provide equally valid inputs for automated ergonomic risk recognition.

The main contributions of this work are twofold: it provides (i) a comparative evaluation of wearable and multi-camera ML systems for estimating the kinematic variables required by the RNLE and calculating the corresponding multipliers (HM, VM, DM, and AM), the recommended weight limit, and the lifting index during lifting activities, and (ii) an assessment of the functional validity of ML data for training AI models to automatically classify the biomechanical risk, demonstrating that these data are comparable to wearable systems and could significantly simplify workplace risk assessments by providing robust and repeatable measurements.

## 2. Materials and Methods

### 2.1. Subjects

We enrolled 13 females and 15 males with a mean age, height, weight, and body mass index of 40.56 ± 14.64 years, 1.71 ± 0.22 m, 74.17 ± 9.57 kg, and 25.29 ± 2.01 kg/m^2^, respectively. None of the individuals had a history of back discomfort, upper or lower limb or trunk surgery, neurological or orthopaedic illnesses, or vestibular system issues. Neither group was included in any clinical pharmaceutical trials. After being given a detailed explanation of the experimental procedure, the participants gave written informed consent for the study, which complied with the Helsinki Declaration and was approved by the local ethics committee (N.0078009/2021). To avoid potential bias, the expected results were not mentioned.

### 2.2. Kinematics Recordings

**Figure 1:**
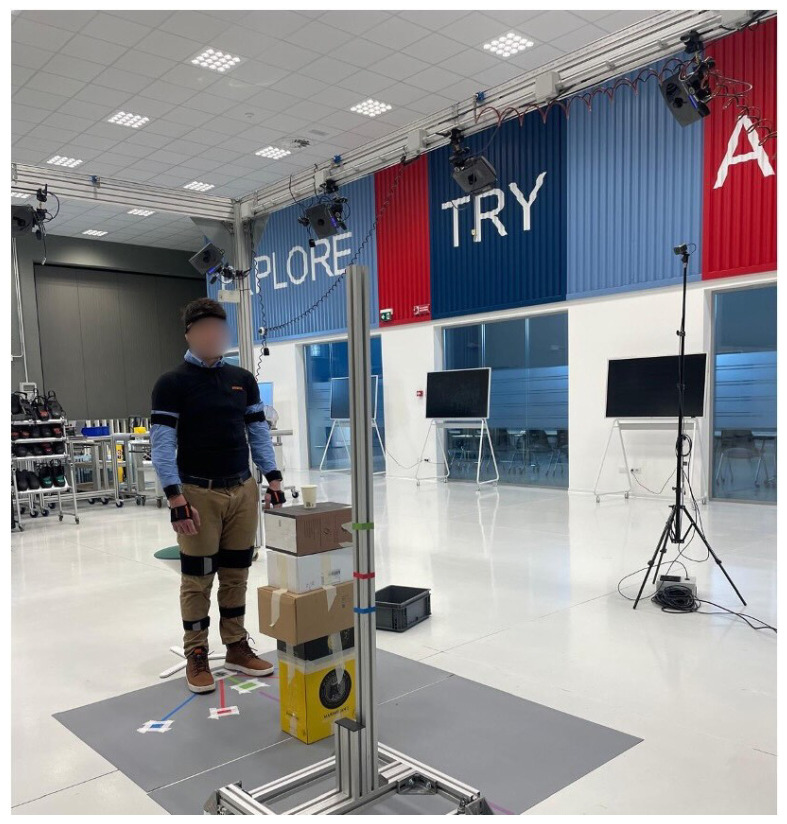
Representation of the experimental setup with a sample subject standing at the center of the calibration field, monitored by infrared cameras and equipped with IMUs.

In this study, we considered only signals that could be recorded without interfering with the physiological motor strategy of the recruited workers during the execution of lifting activities: miniaturized and wirelessly connected IMUs and infrared cameras, as shown in Figure 1.

#### 2.2.1. IMUs

We used Xsens MVN Awinda IMUs (Xsens, Enschede, The Netherlands) to acquire, at a sampling frequency of 60 Hz, the angles and angular velocities of the body joints and the positions and accelerations of the segments. Seventeen IMUs were placed in a whole-body lycra suit in various sizes from M to XXL to monitor the pelvis at the lumbosacral level, the sternum and head, and the foot, shank, thigh, hand, forearm, upper arm, and scapula bilaterally. Measurements of anthropometric data (body height, foot length, shoulder height, shoulder width, elbow span, wrist span, arm span, hip height, hip width, knee height, ankle height, extra shoe sole thickness, and wrist–knuckle distance) were stored in a BodyMeasurement.mvna file and calibration with the subject standing in a T-pose was required to estimate the proportion of the person being tracked and the orientation of the sensors. The recording was executed by using the Xsens MVN Analyze software (Xsens, Enschede, The Netherlands) version 2022.0.2.

#### 2.2.2. Infrared Cameras

Eight infrared cameras with 2 million pixels (SMART-DX Evo 2 System, BTS, Milan, Italy) were arranged on wall brackets around an acquisition volume of approximately 4 m × 3 m × 3 m to ensure at least 50% overlapping coverage. The cameras were used at a sampling rate of 340 Hz to monitor whole-body kinematics by using an ML approach. A calibration procedure allowed for the definition of a global x, y, z axis reference system consistently with what is indicated by the International Society of Biomechanics [33,34]. The resulting residual calibration error was below 0.1 mm, consistent with the manufacturer’s specifications. Infrared (IR) acquisition was executed through the SMART Capture (BTS Bioengineering, Milan, Italy) software version 1.3.3.3 Data acquisition from the IMUs and infrared cameras was synchronized by configuring Xsens MVN Analyze as the master device, which controlled the timing of SMART Capture as the slave system.

### 2.3. Experimental Procedures

The participants performed the lifting tasks while maintaining a neutral standing posture. Each task involved lifting a plastic crate with both hands under three distinct risk conditions, which were established according to the RNLE. These conditions were selected to correspond to reference LI values of 1, 2, and 3, representing increasing levels of biomechanical risk. For each risk condition, Table 1 provides detailed parameters, including the LC, L, H, V, D, A, and F. Additionally, the table lists the respective multipliers applied within the RNLE framework for each parameter. Across all conditions, the hand-to-object coupling was consistently classified as “good,” indicating optimal grip quality during the lifting tasks.

Each participant was required to perform a total of 15 trials and 5 repetitions for 3 risk conditions. The order of each risk condition was randomly assigned.

### 2.4. Data Pre-Processing

#### 2.4.1. Wearable Sensor Network

**Figure 2:**
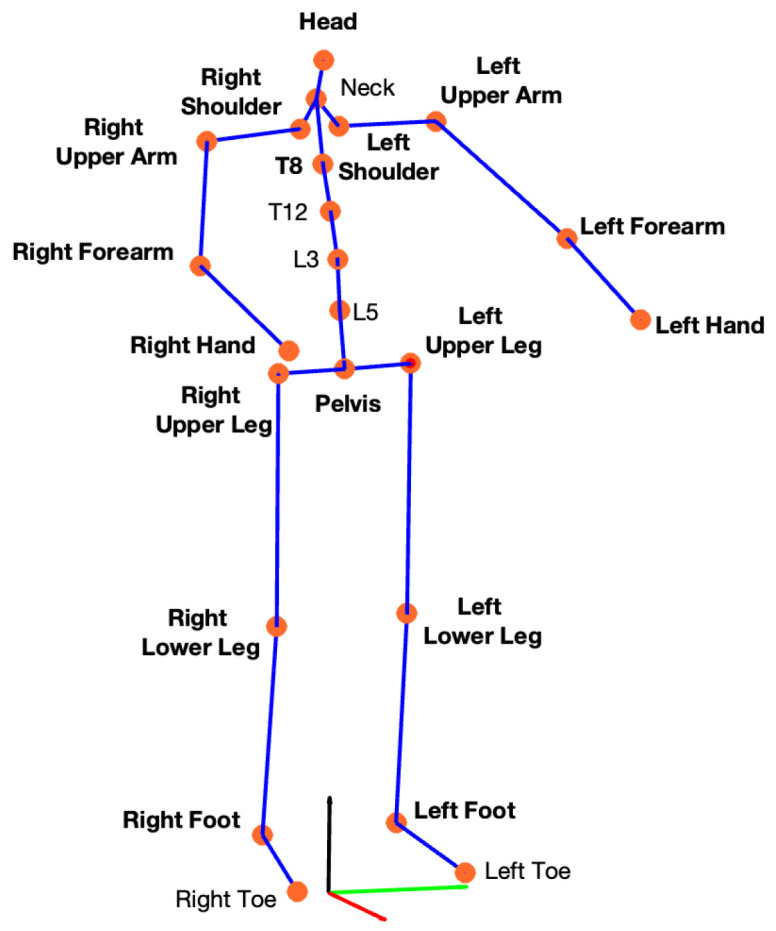
Keypoints extracted using the Xsens protocol. Each of the 17 IMUs was placed on a specific body segment (indicated in bold), the position and orientation of each segment were estimated and are depicted as orange keypoints. An additional 6 body segments were defined to extract the remaining keypoints.

The whole-body kinematics were obtained by using the software Xsens MVN Analyze, Enschede, The Netherlands) version 2022.0.2, which combined the data of all IMUs with advanced biomechanical models to obtain the position and orientation of all human body segments. According to the Xsens protocol, 23 keypoints all over the human body were extracted: 17 from the corresponding sensors placed on the subject and 6 additional segments (Figure 2) estimated by combining the information of the connected segments and the biomechanical model [35].

#### 2.4.2. Markerless

The CapturyStudio software (The Captury GmbH, Saarbrüken, Germany) version 2.9.0 was used to extract 3D skeletons from the IR sequences. For each acquisition, the software pipeline required as an input the 8 IR sequences as .avi files and a configuration file that contained the camera calibration parameters (i.e., EVOCalib.xml). Then, choosing a scaling factor = 1, the acquisition volume was reconstructed.

To extract the whole-body kinematics from IR cameras, the human figure is described by a person-specific model obtained from a sparse set of images, incorporating a kinematic skeleton that defines the model’s degrees of freedom (DoF) where each node represents a joint and the limbs are defined by the orientation and length of the connecting vectors, and a statistical model representing the shape and appearance [26]. To enable real-time pose estimation from multiple views, this approach uses “Sums of 3D Gaussians” (SoGs) for the statistical model. Additionally, the image is represented as a sum of 2D spatial Gaussians that cover consistent color blobs [36]. This method was demonstrated to reach a good balance between accuracy and computational cost.

**Figure 3:**
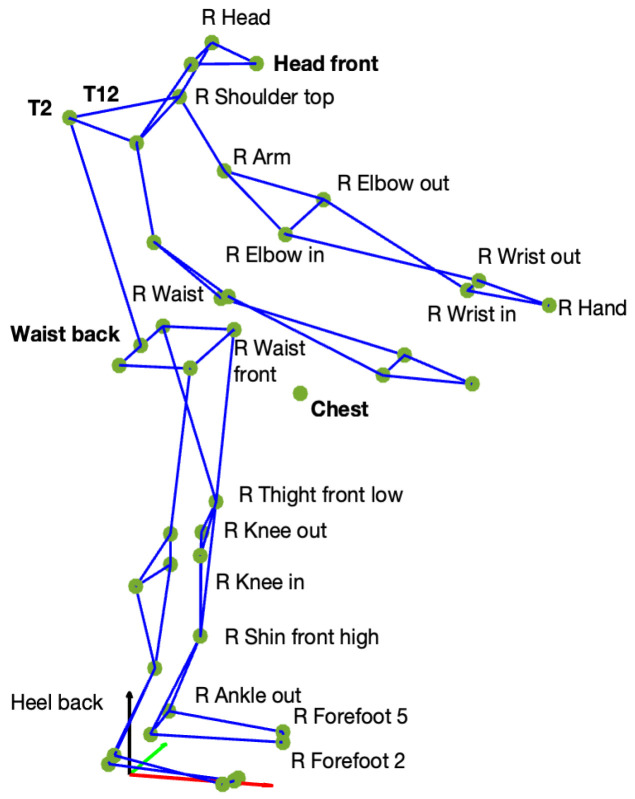
Keypoints extracted from ML data using the Qualisys Sports markerset, specified for the right side (e.g., R Waist). Symmetrical markers were also extracted for the left side (e.g., L Waist), except for the keypoints shown in bold, which are not side-specific (e.g., Waist back). The small circles represent the individual keypoints, and the colored lines indicate the biomechanical connections between them to form the reconstructed skeleton.

Moreover, after the model initialization, a tracking step was performed to execute the feature extraction for the pose estimation process, giving as an output the skeleton in .c3d format. The chosen set of markers, Qualisys Sports (Qualisys, Göteborg, Sweden), enabled the extraction of 42 keypoints (Figure 3).

**Figure 4:**
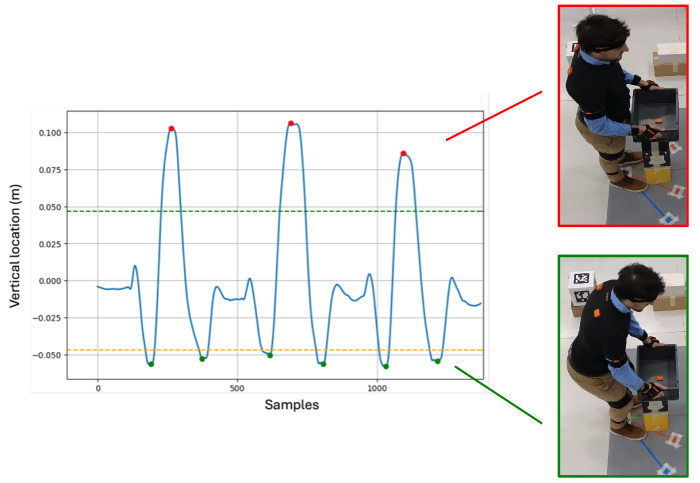
Vertical location of the right wrist during the lifting task. The colored dashed lines represent the threshold (positive in green, negative in orange) used to identify the negative (green dots) and positive (red dots) peaks.

After extracting the whole-body kinematics for both approaches, the vertical location of both the right and left wrist Xsens keypoints were used [18], detecting the beginning of each lifting as the negative peaks, followed by a positive peak that corresponded to the end of the lifting, as shown in Figure 4. Then, the indices corresponding to the beginning and the end of each cycle were stored to match the same segmentation on the ML dataset. Lowering cycles were removed from this analysis.

**Figure 5:**
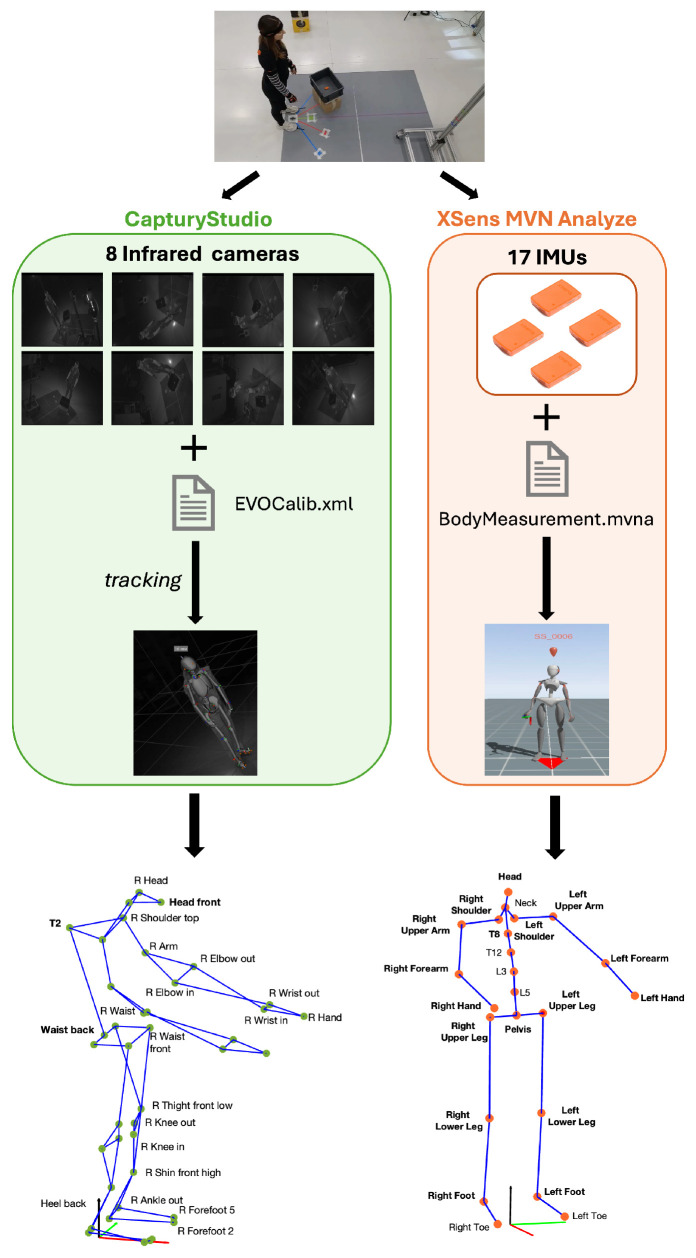
Pipeline of the experimental procedure. For each acquisition, simultaneous recordings of infrared (IR) sequences and IMU signals were performed. In the CapturyStudio software, IR sequences (8) and the calibration file (EVOCalib.xml) enabled the 3D reconstruction of the scene. After a tracking process where a generic skeleton was overlaid on a sample sequence, the subject-specific model of the skeleton was reconstructed. By exporting the skeleton, a graph composed of 42 keypoints/green circles) was obtained. Similarly, in the Xsens MVN Analyze software, the subject-specific model was derived from the scaling of the Xsens Human Body Model according to the subject’s measurements (BodyMeasurement.mvna) and the recording of the 17 IMUs. Then, the 23-keypoint skeleton was exported. Arrows indicate the sequential steps of the processing pipeline.

Figure 5 shows the complete pre-processing pipeline, from the same experimental task.

### 2.5. Data Analysis

The load constant LC was set equal to 23 kg, as requested by the RNLE. The multipliers HM, VM, DM, and AM were calculated using the following equations:

(1) $$\begin{array}{cc} HM & =\frac{0.25}{H(\mathrm{start})}=\frac{0.25}{{\mathrm{Hands}}_{\mathrm{MP}}\left(\mathrm{start}\right)-{\mathrm{Ankles}}_{\mathrm{MP}}\left(\mathrm{start}\right)}, \end{array}$$

(2) $$\begin{array}{cc} VM & =1-0.3\cdot \left|0.75-V(\mathrm{start})\right|, \end{array}$$

(3) $$\begin{array}{cc} DM & =0.82+\frac{0.045}{D}=0.82+\frac{0.045}{\left|V(\mathrm{stop})-V(\mathrm{start})\right|}, \end{array}$$

(4) $$\begin{array}{cc} AM & =1-0.0032\cdot \left|A(\mathrm{stop})-A(\mathrm{start})\right|. \end{array}$$

where, for each cycle,

- *start* and *stop* indicate the beginning and the end of the cycle, respectively.
- ${\mathrm{Hands}}_{\mathrm{MP}}$ is the midpoint between the left and right third knuckles.
- ${\mathrm{Ankles}}_{\mathrm{MP}}$ is the midpoint between the left and right ankles.
- H(start) is the horizontal distance, at the start instant, between the ground projections of ${\mathrm{Hands}}_{\mathrm{MP}}$ and ${\mathrm{Ankles}}_{\mathrm{MP}}$.
- V(start) is the vertical height of ${\mathrm{Hands}}_{\mathrm{MP}}$ above the floor at the start of the cycle.
- *D* is the vertical travel distance, computed as the difference between the heights of ${\mathrm{Hands}}_{\mathrm{MP}}$ at the *stop* and *start* instants.
- A(stop) and A(start) are the asymmetry angles between the subject’s sagittal plane and the centre of the load at the stop and start instants.

Once the variables and multipliers were calculated, the corresponding RWL and LI were estimated according to the RNLE.

It is important to note that, while the Qualisys marker set includes a hand keypoint, the Xsens system only provides wrist markers. Therefore, to estimate the position of the center of the hand in the Xsens dataset, a subject-specific trigonometric approach was applied. This method involved reconstructing the third knuckle position by incorporating the previously measured wrist-to-knuckle distance for each subject [9].

### 2.6. Statistical Analysis

We used the MATLAB (The MathWorks Inc., Natick, MA, USA) software version R2024b to perform the statistical analyses. The Shapiro–Wilk test was used to verify the normal distribution of the data. For each risk condition, and for both the wearable sensor network and ML approaches, we computed the mean values across all trials for each subject for the estimated variables (H, V, D, and A), the corresponding multipliers (HM, VM, DM, and AM), and the resulting RWLs and LIs. Statistical comparisons between the variables, multipliers, RWLs, and LIs estimated using the wearable sensor network and ML methods were carried out for each risk condition. When both data distributions were normal, a paired *t*-test was performed; otherwise, the Wilcoxon signed-rank test was used. The significance level was set at α=0.05.

### 2.7. Integration with Skeleton-Based Action Recognition

The 3D joint coordinates estimated from the wearable sensor network and the ML system were used as input to SkelMamba, a skeleton-based action recognition model used to automatically classify the biomechanical risk levels associated with lifting activities. Each frame sequence was represented as a spatio-temporal skeleton graph, where the nodes correspond to body joints and the edges represent anatomical connectivity. In order to evaluate the ability of the ML model and wearables to generate data as input for human action recognition models capable of classifying the risk level, the SkelMamba model was trained and tested under the same conditions for both the ML and wearable sensor network systems. The model output consisted of discrete risk categories ranging from 1 to 3, corresponding to reference lifting indices (e.g., low, moderate, high).

## 3. Results

### 3.1. Rating of Revised NIOSH Variables

**Figure 6:**
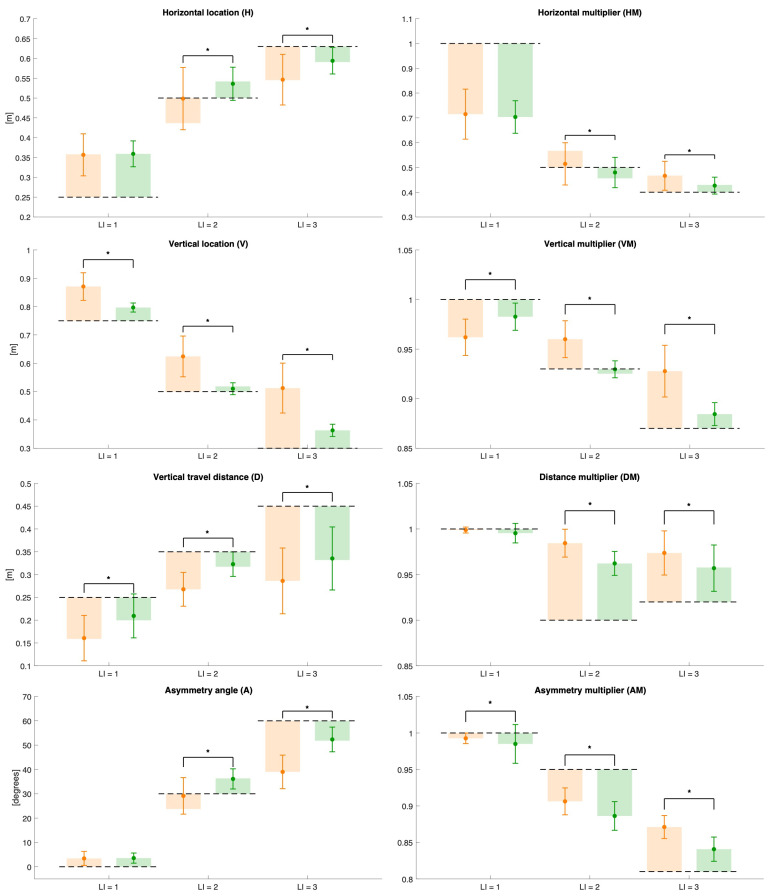
Error plots of the estimated variables (H, V, D, and A) and their corresponding multipliers (HM, VM, DM, and AM) across all risk conditions for both methods (wearable sensor network in orange, ML in green). The dotted lines indicate the reference values, while asterisks denote statistically significant differences between the two approaches. The semi-transparent colored bands represent the mean absolute error.

**Figure 7:**
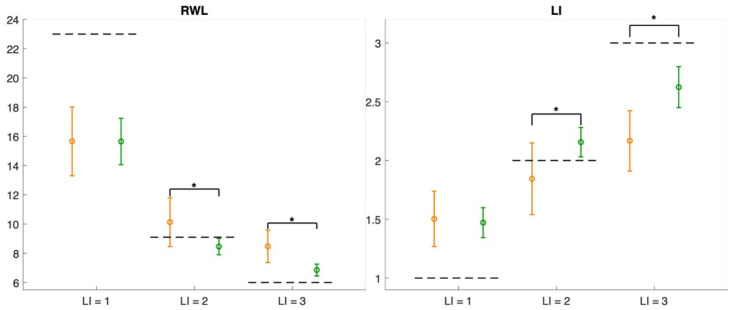
Error plots of the resulting RWL and LI across all risk conditions for both methods (wearable sensor network in orange, ML in green). The dotted lines indicate the reference values, while asterisks denote statistically significant differences between the two approaches.

Figure 6 and Figure 7 present the error plots of the estimated variables, their corresponding multipliers, and the derived indices, RWL and LI, across all the evaluated risk conditions for both measurement methods. For each risk condition, the mean values computed over all subjects are displayed, together with their standard deviations, to represent variability within the sample. The dotted lines correspond to the reference values, serving as benchmarks for comparison.

Overall, the results reveal statistically significant differences between the two measurement methods for the majority of variables and multipliers across the evaluated risk conditions, with the exception of condition 1, where no significant differences were found for the horizontal location and multiplier, the asymmetry angle, the distance multiplier, RWL, and LI.

### 3.2. Automatic Risk Classification Performance

Table 2 shows the results of the integration of the pose estimation methods based on a wearable sensor network or ML with skeleton-based action recognition models. It demonstrates that the extracted joint coordinates were suitable for automatically classifying the lifting index.

Both models reached a high accuracy and a similar loss during training, with minor differences that can be considered negligible given the typical fluctuations during the training. However, the validation metrics showed significant differences between the two systems, highlighting better generalization to the wearable sensor network validation set.

## 4. Discussion

The present study was designed and performed to understand whether ML approaches for human motion reconstruction can be used for instrumental-based biomechanical risk estimation in lifting activities. In particular, the estimate performed on the horizontal and vertical location, vertical travel distance, and asymmetry angle; on the multipliers HM, VM, DM, and AM; and on the RWL and LI with the ML approach was compared with that of the Xsens wearable system consisting of a network of IMU sensors.

The results of this study indicated that the RWL and LI can be calculated using variables estimated with the ML measures under all three risk conditions, with the accuracy of the medium-risk (LI = 2) and high-risk (LI = 3) conditions being higher than that obtained with the wearable-based approach (see Figure 7). Furthermore, for the low-risk (LI = 1) and medium-risk (LI = 2) conditions, an overestimation of the LI is highlighted, while an underestimation under high-risk conditions is reported. This is the opposite of what was estimated for the RWL. This was due to the ability of the ML approach to accurately estimate the multipliers of the RNLE. Figure 6 shows that multipliers are almost always estimated better than what can be achieved with the wearable sensor network. The estimation errors, interpreted as the difference between the mean ± standard deviation and the reference value (indicated by the dashed line), reveal that the largest contributors to RWL underestimation are the horizontal and vertical multipliers of the low-risk condition (LI = 1) and the AM multiplier of both the low-risk (LI = 1) and medium-risk (LI = 2) conditions.

However, the underestimation of the horizontal multiplier may, in fact, have resulted from a discrepancy between the ideal reference value and the actual execution of the task by the participants. Specifically, although a standard horizontal location was expected, the subjects may have performed the task with a different posture or movement strategy. To support this interpretation, the statistical analysis showed no significant differences between the ML and wearable sensor network methods for the horizontal location and, consequently, for the horizontal multiplier.

In the case of the vertical multiplier, the underestimation appeared to be caused by an overestimation of the vertical location (Figure 6). However, the ML method consistently produced values closer to the reference, as further highlighted by the presence of a statistically significant difference between the ML and wearable sensor network methods for the vertical location. This indicates that the ML approach more accurately captures vertical positioning, improving the estimation of the corresponding multiplier.

The scientific literature reports a criticality in the estimation of the asymmetry variable associated with trunk rotation [37]. The results of our study showed a lower accuracy for the ML method compared to wearables, only for the intermediate-risk condition (LI = 2). For the low-risk condition (LI = 1), the accuracies were similar, while for the high-risk (LI = 3) condition, the accuracy of the ML method was higher. This result is particularly encouraging because it indicates that there is a lot of room for the optimization of the ML approach.

On the contrary, the slight overestimation for the high-risk condition (LI = 3) can be attributed to the overestimation of the distance multiplier and the asymmetric multiplier (Figure 6). In the case of the distance multiplier, this resulted from an underestimation of the vertical travel distance, likely due to the overestimation of the hand starting height (V start), which is more challenging to capture accurately in extremely low postures, where the vertical range of motion is larger.

Regarding the asymmetric multiplier, the overestimation may be explained by the accumulation of small errors in the estimation of the sagittal plane, derived from the positions of the left and right pelvic keypoints. These errors become more prominent under demanding movement conditions, such as those observed during high-risk tasks, where a greater trunk rotation and compensation strategies are more common. These errors are particularly impactful for the asymmetric multiplier, whose calculation involves several anatomical landmarks and is therefore highly sensitive to even minor inaccuracies. Furthermore, the discrepancy between the estimated and reference values of the asymmetry angle may not solely reflect inaccuracies in motion tracking, but also differences in task execution. The reference value assumes an idealized posture, whereas the participants may introduce slight torsions or compensations during the lift, even under standardized conditions. Lastly, systematic differences in how the ML and wearable sensor network methods define and detect the pelvic keypoints, due to differing marker sets and estimation pipelines, may further contribute to the observed bias in the asymmetric multiplier.

It is important to note that, under low-risk conditions (LI = 1), both methods exhibited substantial deviations from the reference values, particularly concerning the horizontal location and, consequently, the horizontal multiplier, the recommended weight limit, and the lifting index. The absence of significant differences between the methods at lower risk levels (LI = 1) suggests better consistency, while higher-risk conditions showed greater divergence. One possible reason for the absence of a statistically significant difference in the risk condition with LI = 1 lies in the different ways in which the two systems reconstruct the skeleton. The wearable system reconstructs the joints based on a kinematic model that can be scaled according to the anthropometric measurements entered for each subject, while the markerless system does so independently of anthropometric measurements by assigning a model to the image. This difference may be amplified in dynamic tasks where postures change more, as in the case of risk conditions LI = 2 and LI = 3. Furthermore, from a statistical point of view, the measured variability makes it unlikely that there is any difference between two means that are very close to each other. Consistent significant deviations from reference values point to systematic biases that warrant further investigation. Furthermore, these results indicate that agreement between the measurement methods varies depending on the risk condition and variable analyzed. Conversely, the ML method consistently demonstrated closer conformity to the reference benchmarks across all conditions and presented a reduced variability, as evidenced by lower standard deviations relative to the wearables. These observations suggest that, although the two methods differ in their estimations overall, the ML method provides more precise and reliable measurements, especially outside of low-risk conditions (LI = 1).

The possibility of estimating the input variables of several methods for biomechanical risk estimations in manual material handling activities with opto-electronic or wearable sensor networks is known [1,18,38,39], but minimal evidence is available on the estimation through ML approaches [40]. By accurately estimating and computing the RNLE variables, RWL, and LI, a wearable sensor network can reliably evaluate the ergonomic risk associated with lifting tasks. Nevertheless, several enhancements are required, particularly lowering the measurement variability and boosting the sensitivity [1]. Our results confirm that instrumental methods are increasingly ready for a quantitative assessment of the biomechanical risk in heavy lifting activities, as also foreseen by a recent pre-standard (CWA).

Specifically for heavy lifting tasks, ML approaches can even provide a greater accuracy and precision than wearable-based methods [18,38].

A current limitation is represented by the fact that we tested a complex ML solution in terms of the technology (infrared), number of cameras (eight), and computational and time-consuming aspects. However, the evaluation of the feasibility and accuracy of this system serves as a precursor to more portable configurations, such as single-depth or RGB-based systems. A further limitation is represented by the fact that, in an analogy with what has been conducted in other studies, it was not possible to make an inference between the measurements carried out with the wearable sensor network and ML systems and the reference values. This was because, although the lifts were designed to be performed in a standardized manner, the recruited subjects inevitably performed them somewhat differently. Another limitation of the study is associated with the fact that no quantitative analysis of the subjects’ differences in performing the tasks was conducted.

A first development of the present study could be to investigate the accuracy of the ML system by considering all the combinations of cameras with a number less than eight until the self-occlusion problem makes the measurement no longer truthful. In this way, there would be the possibility of simplifying the monitoring setup with the same accuracy and precision of estimation. Furthermore, it could be extremely useful to compare the measurements of the ML system with those obtained with conventional low-cost video cameras. This would reduce the cost of the monitoring system. For instance, ML systems, such as the Kinect series, are low-cost compared to wearable sensor networks.

A natural evolution in line with the digitalization underway in the workplace would be to implement artificial intelligence algorithms to optimize and automate risk classification. In fact, skeleton-based action recognition models could be a useful tool to recognize motor pattern during working activities and to assign each movement a risk factor according to the ergonomic standards. These models can take as the input the sequence of the human body joints moving in time, seeing it as a graph structure. Since this skeleton can be extracted with several systems (ML, wearable sensor network, or RGB camera-based systems), a valuable insight could be the study of the impact of each of these pose estimation methods in the direct risk level classification. In the future, a complete tool provided with all the ergonomic standard classifications (NIOSH, OCRA, etc.) could be developed to assess the biomechanical risk starting from video-based data. Further efforts should be made to develop the 3D reconstruction of complex lifting activities, such as composite or variable lifting activities [41,42,43]. Complex lifting tasks consist of lifting with different geometries and/or loads and represent more flexible actions than a mono-task. Finally, the challenges associated with privacy concerns must be deepened for wider use [22].

## 5. Conclusions

The results of this study show that the LIs computed from the two acquisition methodologies were close for all the activities analyzed, but that the ML approach showed a higher precision. As for the integration with skeleton-based AI models, the ML method showed a comparable performance in predicting the lifting index with a high accuracy. However, bigger differences on the validation metrics showed that the wearable sensor network produced more coherent and discriminative data. For this reason, the ML method seems very promising in promoting an instrumental-based biomechanical risk assessment in lifting tasks while maintaining a high accuracy and affordable costs.

## Figures and Tables

**Table 1:** For each lifting task from T_1_ to T_3_, values of the load weight (L), horizontal (H) and vertical (V) locations, vertical travel distance (D), asymmetry angle (A), lifting frequency (F), and hand-to-object coupling (C) and the corresponding multipliers and recommended weight limit (RWL) are given. Bold text highlights variable labels; italics indicate directly measured geometric parameters; shaded cells are used only to improve readability.

**Lifting task**	T1	T2	T3
**Reference lifting index**	**1**	**2**	**3**
**Load constant LC (kg)**	23	23	23
**Load L (kg)**	23	18.2	18
**Recommended weight limit RWL**	23	9.1	6
**Horizontal location H (cm)**	*25*	*50*	*63*
**Horizontal multiplier HM**	1	0.50	0.40
**Vertical location V (cm)**	*75*	*50*	*30*
**Vertical multiplier VM**	1	0.93	0.87
**Vertical travel distance D (cm)**	*25*	*35*	*45*
**Distance multiplier DM**	1	0.90	0.92
**Asymmetry angle A (°)**	*0*	*30*	*60*
**Asymmetric multiplier AM**	1	0.95	0.81
**Frequency F (lift/min)**	≤0.2	≤0.2	≤0.2
**Frequency multiplier FM**	1	1	1
**Coupling factor C**	good	good	good
**Coupling multiplier CM**	1	1	1

**Table 2:** Comparison of multiclass performances between the two methods.

Metric	Markerless (ML)	Wearable Sensor Network
Train Accuracy	0.9954	0.9997
Train Loss	0.298	0.292
Val Accuracy	0.832	0.933
Val Precision	0.826	0.947
Val Recall	0.832	0.933
Gap	0.163	0.066

## Data Availability

The datasets presented in this article are not readily available because the data are part of an ongoing study. Requests to access the datasets should be directed to the corresponding author.
